# Supplementary material for: Clarifying the quantum mechanical origin of the covalent chemical bond
Source: Nat Commun. 2020 Sep 29;11:4893. doi: 10.1038/s41467-020-18670-8 (PMC7524788; doi:10.1038/s41467-020-18670-8)
Supplement: Supplementary file 1 — Supplementary Information [file 41467_2020_18670_MOESM1_ESM.pdf]

# Clarifying the Quantum Mechanical Origin of the Chemical Bond

Daniel S. Levine, Martin Head-Gordon

## Supporting Information

# Supplementary Notes

## Supplementary Note 1

While the contraction step[1] is summarized in the Methods section, we wish to enlarge here on how the contraction wavefunctions are determined. As the ALMO-EDA scheme is based on a series of variationally optimized wavefunctions beginning from separated fragments, the variational constraints used to define those intermediate wavefunctions are given by the portion of Hilbert space made available to the molecule in which to find the variationally optimal energy. We therefore must define a set of virtual orbitals that augment the Hilbert space already occupied by the occupied orbitals and these virtuals must allow for contraction (and nothing else). We base the selection of these virtuals on fragment electric response functions (FERFs).[2] That is, we seek those virtual orbitals that exactly define the response of the density to a particular pertubation. In the case of polarization functions, these are electric fields. In the case of contraction, the perturbation is an infinitessimal change in the nuclear charge, as increasing the nuclear charge would cause the density to uniformly contract toward the nucleus. With superscripts as derivatives, $\Delta$ is an orbital perturbation, $Z$ is a nuclear charge perturbation, $V_{NE}$ is the nuclear-electron attraction, and $P$ is the density, then the monopole FERFs may be solved by the coupled perturbed-SCF linear equation:

$$E^{\Delta\Delta} \cdot \Delta^{Z} = -E^{\Delta Z} = -V_{NE}^{Z} \cdot P^{\Delta}$$

The result is precisely one virtual orbital per occupied orbital which describes that occupied orbitals response to such a perturbation and therefore describe contraction. By defining the contraction in this way, we obtain the desired effect with a well-defined basis set limit (increasing the size of the basis does not increase the number of virtual response orbitals, merely improves the description of the response). Moreover, these contraction orbitals are solely a property of the fragment of interest, regardless of that fragment's chemical environment.

## Supplementary Note 2

A comparison of the orbital contraction definition employed here and those of other authors may prove enlightening. Hiberty and co-workers[3,4] define contraction by comparing the squares of orbital coefficients of specifically selected atomic orbitals (which are more or less diffuse) between the free atom and the atoms in the final wave function. Our approach,[1] on the other hand, determines a set of orbitals within the full span of the virtual space, one for each occupied orbital, which exactly describes the response of the occupied orbitals to a perturbation of the nuclear charge (or equivalently, placing a point charge at the nucleus). Critically, while Hiberty et al. compare the coefficients of orbitals with different spatial extent in order to measure contraction in real-space, our method interrogates contraction in energy-space. That is, we make no assertion about changes to the real-space size of orbitals due to contraction (although such an analysis could be done by comparing the real-space density before and after the variational contraction step), only the energetic stabilization attained by allowing contraction to take place. We weigh the importance of contraction only inasmuch as it lowers the total energy, not by how it changes the physical shape of orbitals. Moreover, the essential purpose of determining the response orbitals is to search out the "best" (in an energetic sense) description of a contracting orbital from the entire virtual span, while Hiberty and co-workers manually assign a specific atomic orbital to that purpose. In our approach, a well-defined basis set limit is achieved in which a larger and larger basis set will produce a better and better representation of the contraction virtual orbitals. While the orbitals chosen by Hiberty and co-workers are certainly excellent, physically reasonable guesses for contraction orbitals, they may contain contributions from other physical processes, such as polarization, which may explain why there are some specific examples where the two methods do not agree on the degree of contraction present. On the whole, however, the methods concur that the contraction phenomenon is not universal in bond formation. Ruedenberg and co-workers[5] appears to define contraction with respect to quasi-atoms, which are constructed from the final molecular wave function, and free atoms. Specifically, after the quasi-atoms are determined, the orthogonal complement of the original orbital span within the quasi-atom basis is found and described as "intra-atomic deformation", which includes radial contraction. The energy of this deformation can then be computed. We believe differences between our findings and Ruedenberg's with respect to contraction are due to how these different reference states (in Ruedenberg's case the quasi-atoms, in ours, the response orbitals) are defined.

## Supplementary Note 3

There are a variety of conceivable definitions to employ for the covalent step in our approach. We will see that all reasonable choices are qualitatively similar to the results discussed in the text. We begin by defining a few states:

(A) Isolated fragments: the fragments of the bond in their geometrically and electronically relaxed forms.

(B) Geometrically prepared fragments: the fragments of the bond distorted to adopt their geometry in the bond

(C) Prepared fragments: the fragments of the bond distorted to the geometry in the bond and electronically prepared (that is, the alpha density is unchanged and the beta hole has been optimized within this span, i.e. "rehybridized")

(D) Heitler-London with geometrically prepared fragments: the two-configurational, interacting bonded system employing identical orbitals to the geometrically prepared fragments

(E) Heitler-London with prepared fragments: the two-configurational, interacting bonded system employing identical orbitals to the prepared fragments

In this (and previous[6]) work, we term the preparation step to be the difference between states (A) and (C) and the covalent step to be between states (C) and (E). We opt for the state (C), as described above, because the geometric preparation can induce some orbital rehybridization changes in some systems but not others. For example, a planar methyl radical is $sp^2$ hybridized while pyramidal methyl is $sp^3$ hybridized. Hence the geometry change induces an electronic change. In contrast, the amide radical enjoys no such hybridization change during its geometric deformation, making these systems difficult to compare. Once we have chosen the orbitals, they must be identical with those of the Heitler-London wave function, and so (E) is the correct choice.

An alternative is to ignore this rehybridization and use precisely the orbitals of the geometrically prepared fragments (B) to form the TCSCF wave function (D). One might also consider defining the covalent step as the difference between steps (A) and (D) or (A) and (E) and having no preparation step at all. We give the results for several of these differences below in the example of butane (see Supplementary Figure 1). The crucial point is that all 4 possible definitions of the covalent step show an increase in the KE of butane at equilibrium ($\sim 1.55$Å): (C)$\rightarrow$(E) as used in the main manuscript, and the alternatives (B)$\rightarrow$(D), (A)$\rightarrow$(D), and (A)$\rightarrow$(E).

(A)$\rightarrow$(B) and (A)$\rightarrow$(C) are both reasonable definitions of preparation and produce qualitatively similar curves. Along the same lines, (B)$\rightarrow$(D) and (C)$\rightarrow$(E) are both reasonable and essentially similar definitions for a post-preparation covalent step. Less convincingly, one can also skip the preparation step and consider (A)$\rightarrow$(D) and (A)$\rightarrow$(E) for an aggregated covalent bond formation step, but even these choices are again qualitatively similar in Supplementary Figure 1 to our preferred choice of (C)$\rightarrow$(E) for the covalent step. We conclude that kinetic energy increases for covalent bond formation at equilibrium in butane for all possible definitions.

Nonetheless, we do not advocate including the geometric changes in the same term as the covalent changes as it necessarily contaminates the physics at play. This is easily seen when considering these states in the butane radical cation (see Supplementary Figure 2). The geometric changes (A)$\rightarrow$(B) alone account for the substantially negative total kinetic

energy changes, (A)→(E), observed at equilibrium ($\sim$ 2.0Å) for the butane radical cation. Including these geometry-driven changes in the covalent step swamps the kinetic energy increasing covalent changes. By contrast, when these geometric changes are not included, that is for (B)→(D) and (C)→(E) we again see a similar effect of the covalent step to slightly increase the kinetic energy associated with covalent bond formation in butane radical cation near equilibrium.

## Supplementary Note 4

An investigation with heavier-than-hydrogen groups[7] was confounded by the fact that multiple, rather than single bonds were investigated. As was pointed out in the 1970's, first row $\pi$-bonds are expected to behave much like hydrogen $\sigma$-bonds due to the absence of inner electrons or radial nodes and in these strongly bound systems will obscure the fact that the $\sigma$ bond in these systems are fundamentally different than the $H_2$ $\sigma$-bond.[8,9] The only single-bonded species in that study was $F_2$ which showed qualitatively identical results to what was obtained here (kinetic energy increased significantly on bringing together two F atoms to equilibrium, see Figure 12 of Schmidt et. al[7]); no comment on this fact was made in that study, as these authors favored a quasi-atomic reference state derived by a procedure from the molecular density, as opposed to atomic fragments as we advocate here. Another study corroborated these qualitative data with other examples, but did not connect those results to the conclusions presented here.[10]

# Supplementary Figures

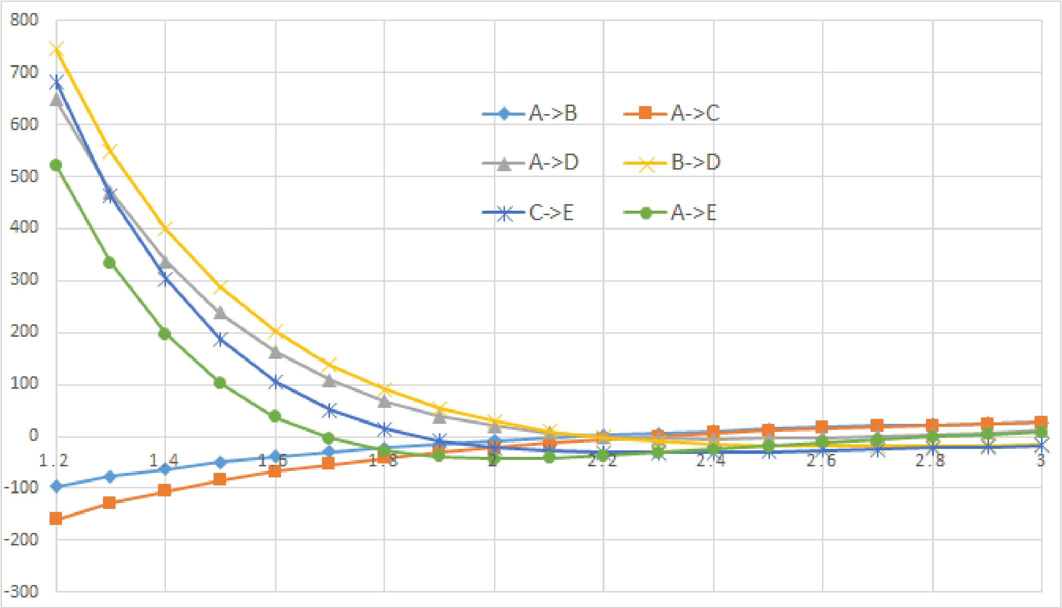


**Supplementary Figure 1.** Butane kinetic energy decomposition (energy in kcal/mol, bond length in Å) with alternative state definitions.

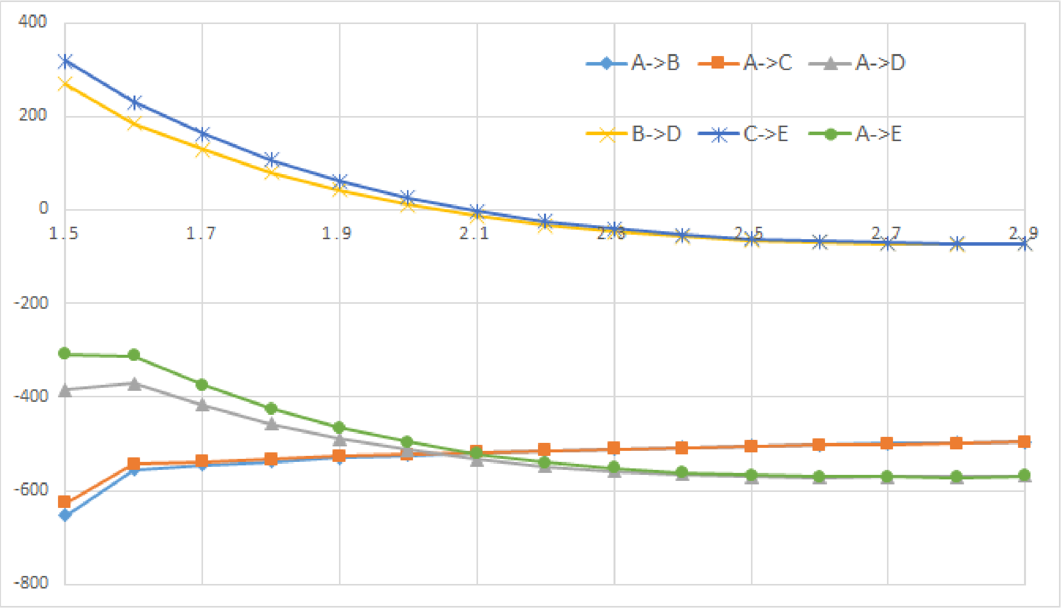


**Supplementary Figure 2.** Butane radical cation kinetic energy decomposition (energy in kcal/mol, bond length in Å) with alternative state definitions.

## EDA/TDA Dissociation Curves

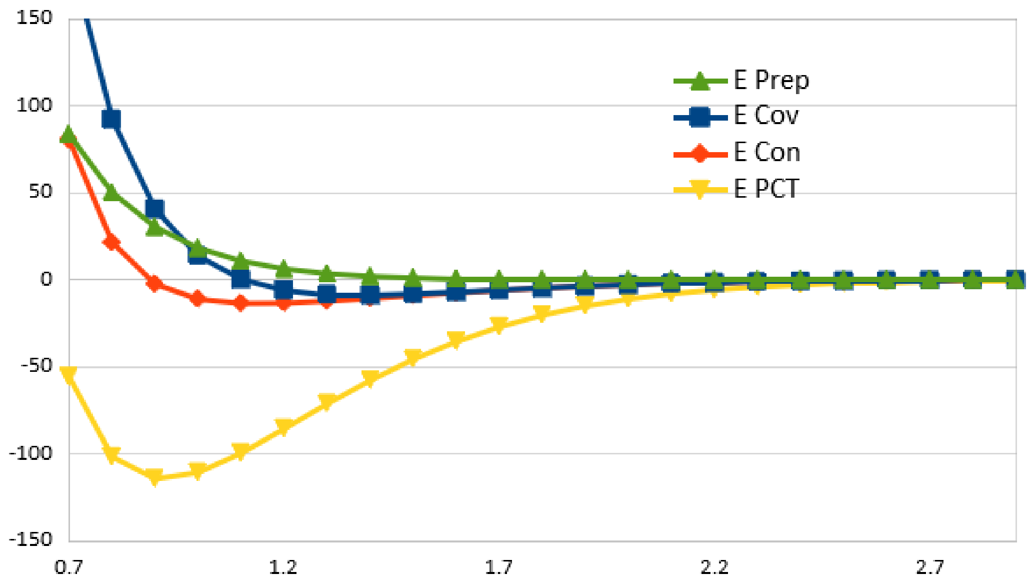


**Supplementary Figure 3.** H−F total energy decomposition (energy in kcal/mol, bond length in Å).

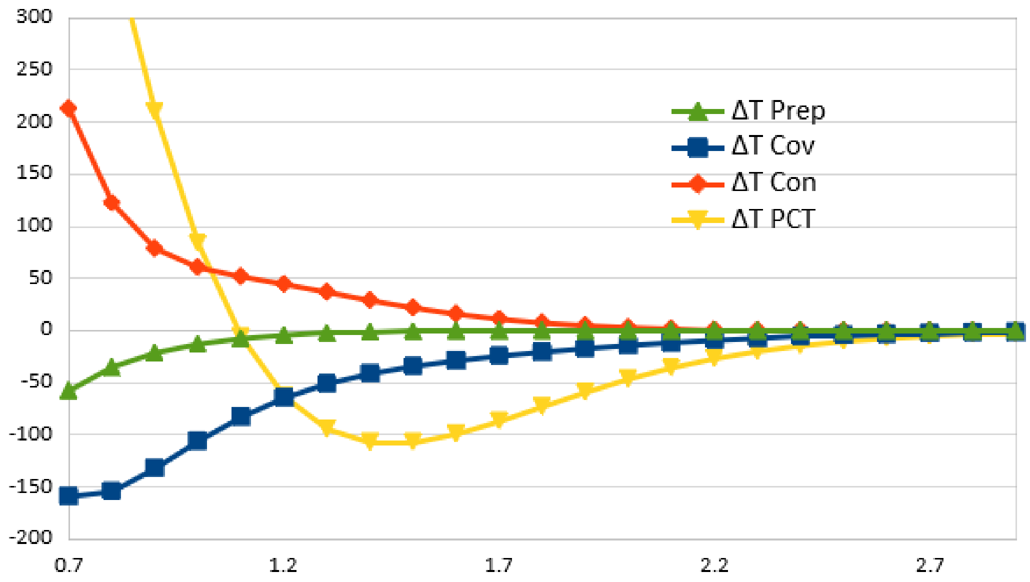


**Supplementary Figure 4.** H−F kinetic energy decomposition (energy in kcal/mol, bond length in Å).

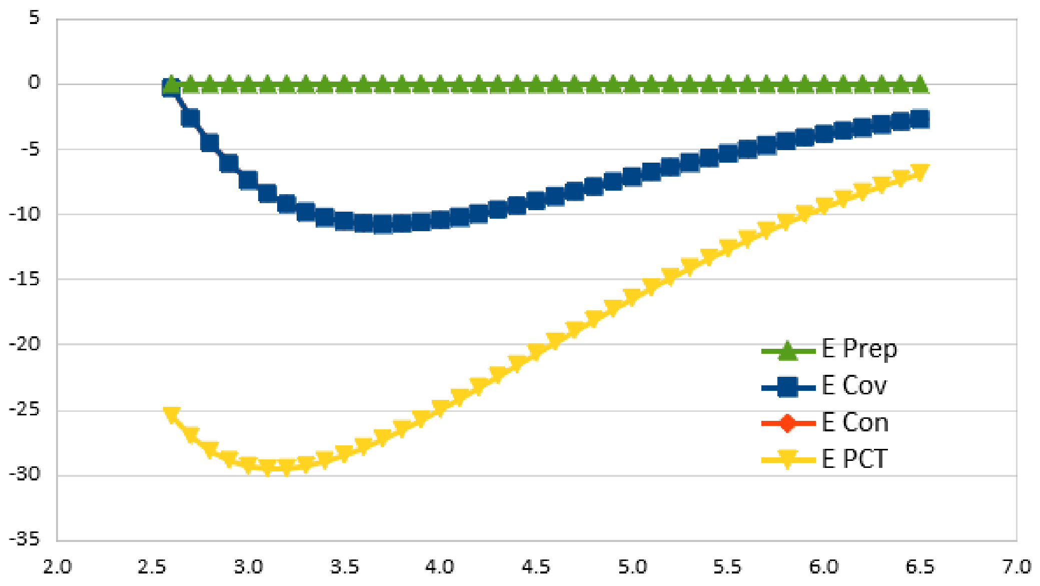


**Supplementary Figure 5.** $[Li{-}Li]^{+}$ total energy decomposition (energy in kcal/mol, bond length in Å).

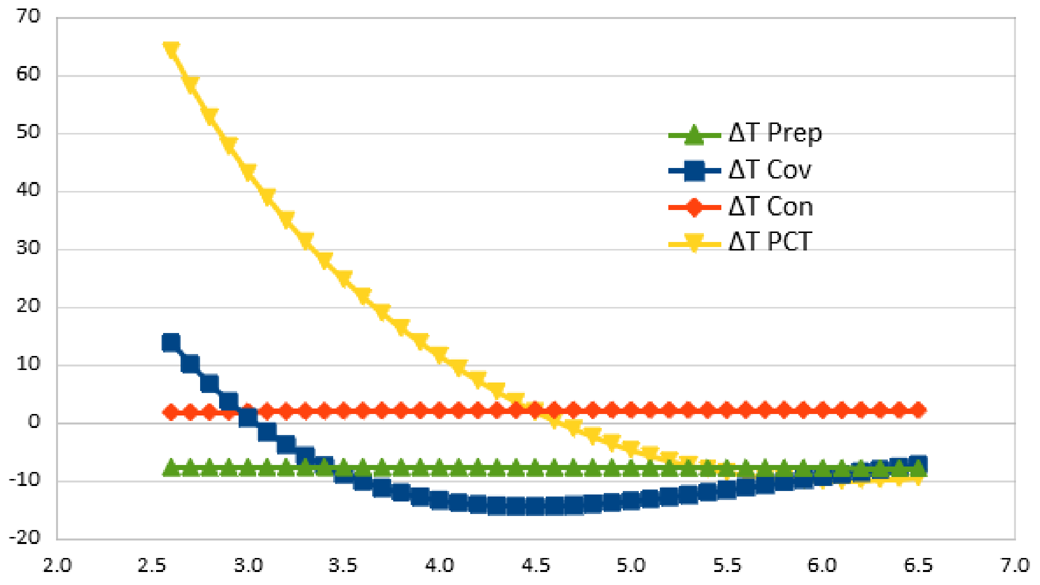


**Supplementary Figure 6.** $[Li{-}Li]^{+}$ kinetic energy decomposition (energy in kcal/mol, bond length in Å).

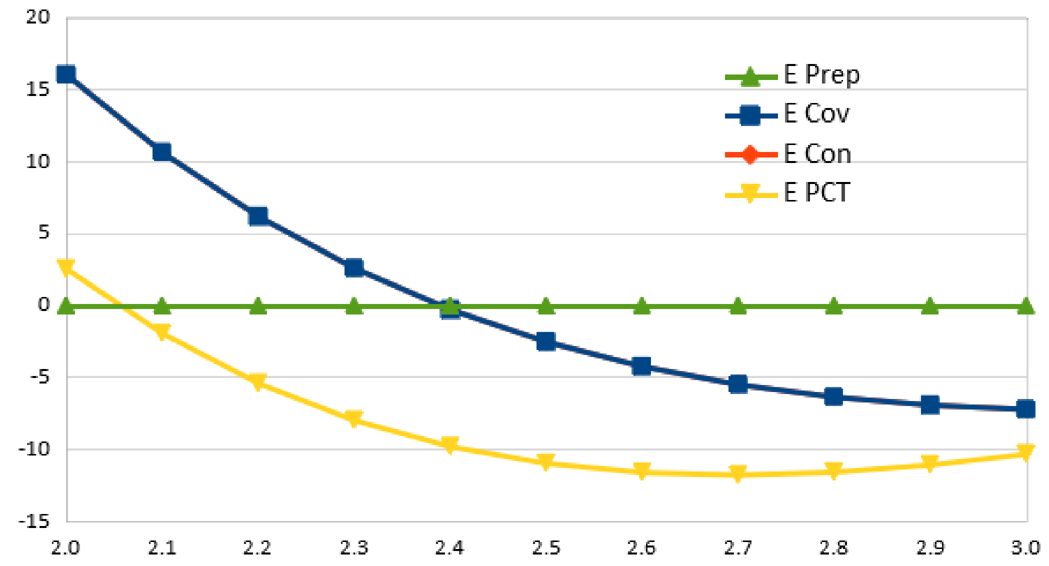


**Supplementary Figure 7.** Li−Li total energy decomposition (energy in kcal/mol, bond length in Å).

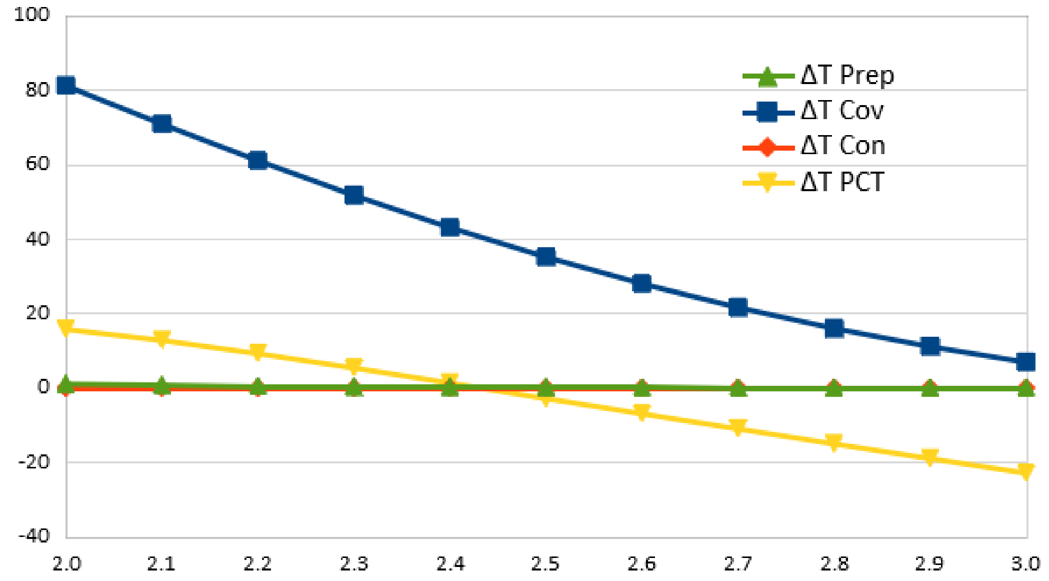


**Supplementary Figure 8.** Li−Li kinetic energy decomposition (energy in kcal/mol, bond length in Å).

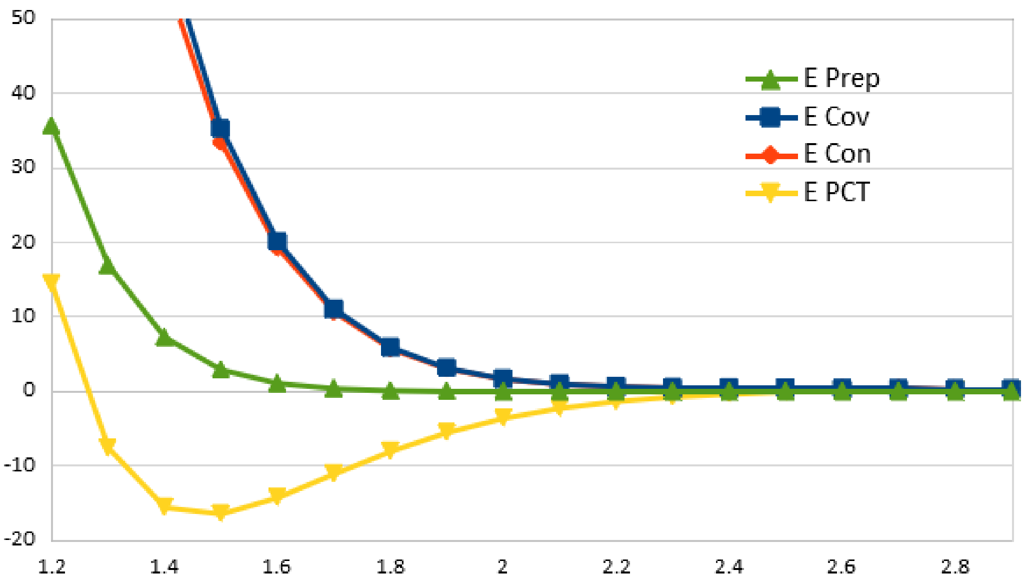


**Supplementary Figure 9.** F−F total energy decomposition (energy in kcal/mol, bond length in Å).

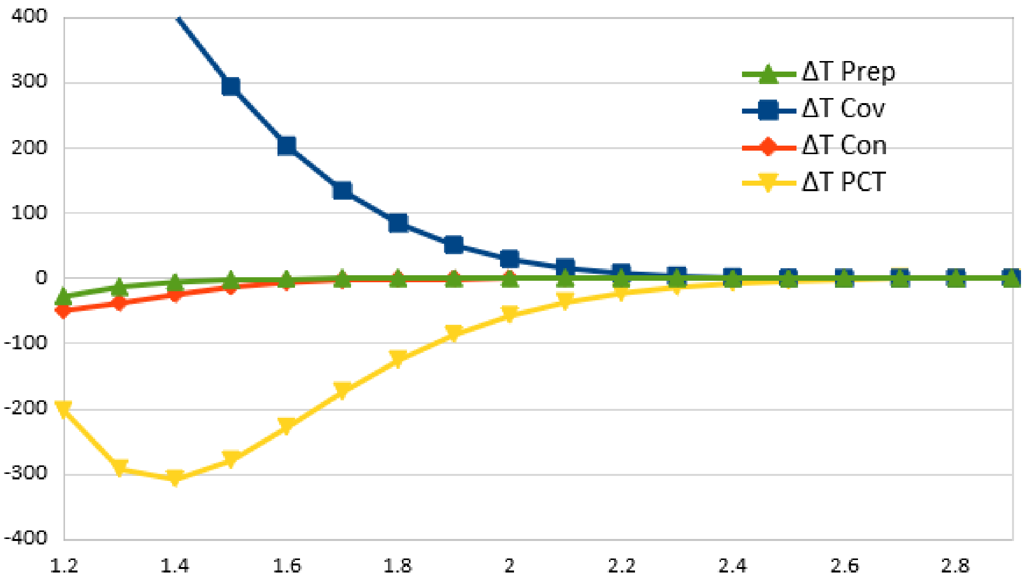


**Supplementary Figure 10.** F−F kinetic energy decomposition (energy in kcal/mol, bond length in Å).

Our energy decomposition analysis, which focuses on determining the physical origin of lowering the total energy, indicates that quantum mechanical wavefunction interference of frozen, ground-state fragments (i.e. $\Delta E_{\mathrm{Cov}}$) is the origin of normal covalent bonding,[11] but

that total energy stabilization need not be accompanied by a corresponding lowering of kinetic energy, particularly in bonds between groups containing core electrons. This lack of correlation can be seen in Figure 11

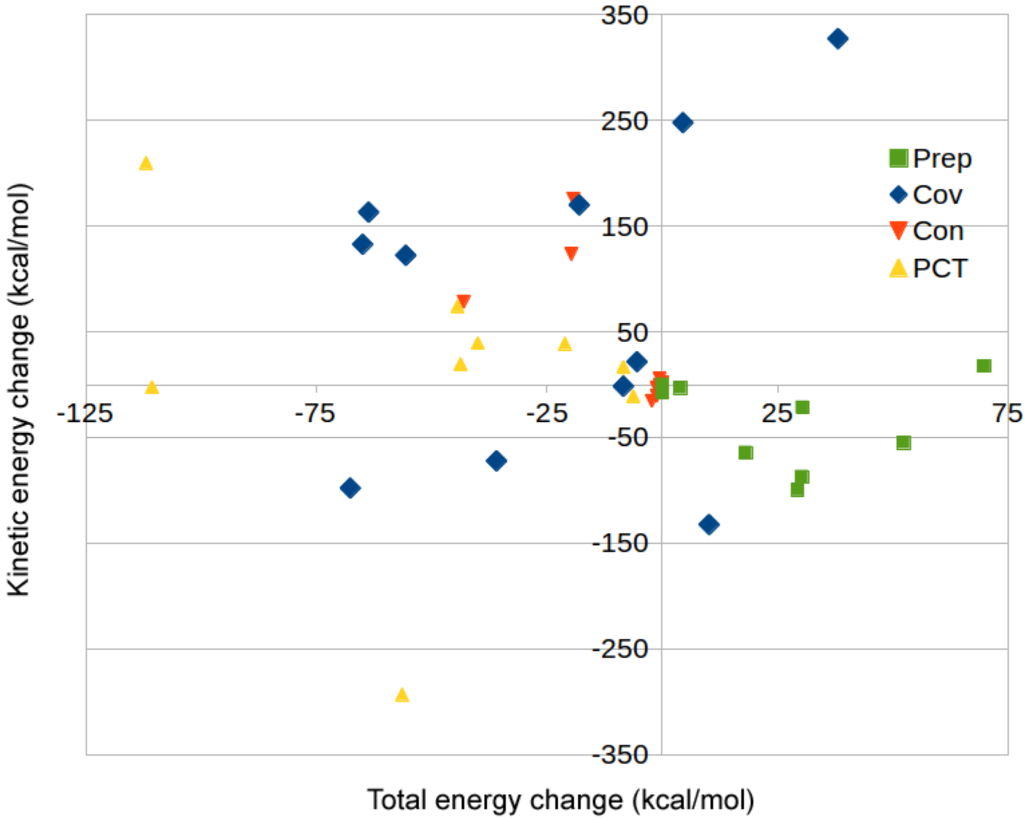


**Supplementary Figure 11.** Scatter plot of kinetic energy changes $\Delta T$ vs. total energy changes $\Delta E$ for each component in the EDA. Prep total energies are guaranteed to lie in the right half plane. $\Delta E_{\text{Con}}$ and $\Delta E_{\text{PCT}}$ are guaranteed in the left half plane. Critically, there is no correlation between total energy changes and kinetic energy changes at the covalent stage (blue diamond data points).

# Supplementary Tables

## Equilibrium data

| | $\Delta T_{\text{Prep}}$ | $\Delta T_{\text{Cov}}$ | $\Delta T_{\text{Con}}$ | $\Delta T_{\text{PCT}}$ | $\Delta E_{\text{Prep}}$ | $\Delta E_{\text{Cov}}$ | $\Delta E_{\text{Con}}$ | $\Delta E_{\text{PCT}}$ |
|---|---|---|---|---|---|---|---|---|
| $H_2^+$ | | -71.4 | 98.3 | 34.7 | | -33.7 | -16.6 | -14.0 |
| $H_2$ | | -97.9 | 175.6 | 16.8 | | -67.6 | -19.3 | -8.4 |
| HF | -21.6 | -132.4 | 78.5 | 209.7 | 30.4 | 10.2 | -43.0 | -111.9 |
| $Li_2^+$ | -7.6 | -1.6 | 1.9 | 38.8 | 0.0 | -8.4 | 0.0 | -21.1 |
| $Li_2$ | 0.1 | 21.7 | 0.0 | -10.9 | 0.0 | -5.5 | 0.0 | -6.3 |
| $F_2$ | -3.1 | 327.2 | -15.3 | -293.2 | 3.9 | 38.1 | -2.3 | -56.4 |
| $H_5C_2-C_2H_5$ | -87.1 | 163.1 | 5.7 | 19.4 | 30.3 | -63.7 | -0.6 | -43.7 |
| $H_3C-CH_3$ | -99.5 | 113.4 | -0.7 | 74.0 | 29.4 | -64.9 | -0.5 | -44.4 |
| $H_3C-OH$ | -54.9 | 169.9 | -3.3 | -2.5 | 52.3 | -18.0 | -1.2 | -110.6 |
| $H_3C-SiH_3$ | -64.4 | 122.3 | -1.6 | 39.0 | 18.1 | -55.6 | -0.3 | -40.0 |
| $F-SiF_3$ | 17.6 | 247.8 | -10.6 | -124.3 | 69.8 | 4.5 | -1.2 | -215.5 |

**Supplementary Table 1.** EDA and TDA data (kcal/mol) for various molecule at equilibrium using HF determinants.

| | $\Delta T_{\text{Prep}}$ | $\Delta T_{\text{Cov}}$ | $\Delta T_{\text{Con}}$ | $\Delta T_{\text{PCT}}$ | $\Delta E_{\text{Prep}}$ | $\Delta E_{\text{Cov}}$ | $\Delta E_{\text{Con}}$ | $\Delta E_{\text{PCT}}$ |
|---|---|---|---|---|---|---|---|---|
| $H_2$ | 0.0 | -100.0 | 175.2 | 28.3 | 0.0 | -76.0 | -19.6 | -16.4 |
| HF | -21.5 | -137.4 | 99.4 | 198.0 | 30.0 | -4.5 | -34.6 | -141.6 |
| $F_2$ | -8.0 | 472.7 | -1.7 | -395.4 | 10.1 | 54.6 | -3.4 | -111.2 |
| $H_3C-CH_3$ | -84.5 | 104.7 | 3.2 | 74.6 | 35.3 | -82.7 | -0.6 | -64.1 |

**Supplementary Table 2.** EDA and TDA data (kcal/mol) for various molecule at equilibrium using DFT ($\omega$B97X-D) determinants.

| | $E_{initial}$ | $T_{initial}$ | $E_{final}$ | $T_{final}$ | $\Delta E$ | $\Delta T$ | $E/T_{initial}$ | $E/T_{final}$ |
|---|---|---|---|---|---|---|---|---|
| $H_2^+$ | -0.499821176 | 0.499305885 | -0.602000073 | 0.585833414 | -64.3 | 61.7 | -1.0010 | -1.0078 |
| $H_2$ | -0.999642352 | 0.998611758 | -1.151602179 | 1.1491063 | -95.4 | 94.4 | -1.0010 | -1.0022 |
| $Li_2^+$ | -14.66905178 | 14.6569843 | -14.71610882 | 14.71924412 | -29.5 | 39.1 | -1.0008 | -0.9998 |
| $Li_2$ | -14.86536469 | 14.86559353 | -14.88410614 | 14.88275612 | -11.8 | 10.8 | -1.0000 | -1.0001 |
| HF | -99.90200593 | 99.88675188 | -100.0843127 | 100.1006191 | -114.4 | 134.2 | -1.0002 | -0.9998 |
| $F_2$ | -198.8043695 | 198.774892 | -198.8309094 | 198.7998317 | -16.7 | 15.6 | -1.0001 | -1.0002 |
| $H_3C-CH_3$ | -79.14750419 | 79.12627196 | -79.27576728 | 79.2652207 | -80.5 | 87.2 | -1.0003 | -1.0001 |
| $H_5C_2-C_2H_5$ | -157.249217 | 157.2121512 | -157.3730023 | 157.372004 | -77.7 | 100.3 | -1.0002 | -1.0000 |
| $CH_3OH$ | -114.9909369 | 114.9673127 | -115.114417 | 115.1414251 | -77.5 | 109.3 | -1.0002 | -0.9998 |
| $H_3C-SiH_3$ | -330.214808 | 330.1827407 | -330.3388087 | 330.3347773 | -77.8 | 95.4 | -1.0001 | -1.0000 |
| $F-SiF_4$ | -686.9589599 | 686.8206356 | -687.1859641 | 687.0284965 | -142.4 | 130.4 | -1.0002 | -1.0002 |

**Supplementary Table 3.** Total energies and total kinetic energies satisfy the Virial theorem at equilibrium

# Supplementary References


[1] Levine, D. S.; Head-Gordon, M. Quantifying the Role of Orbital Contraction in Chemical Bonding. *J. Phys. Chem. Lett.* **2017**, *8*, 1967–1972.

[2] Horn, P. R.; Head-Gordon, M. Polarization Contributions to Intermolecular Interactions Revisited with Fragment Electric-Field Response Functions. *J. Chem. Phys.* **2015**, *143*, 114111.

[3] Hiberty, P. C.; Ramozzi, R.; Song, L.; Wu, W.; Shaik, S. The physical origin of large covalent-ionic resonance energies in some two-electron bonds. *Faraday Discuss.* **2006**, *135*, 261–272.

[4] Shaik, S.; Danovich, D.; Galbraith, J. M.; BraÃ¯da, B.; Wu, W.; Hiberty, P. C. Charge-Shift Bonding: A New and Unique Form of Bonding. *Angew. Chem. Int. Ed.* **2020**, *59*, 984–1001.

[5] West, A. C.; Schmidt, M. W.; Gordon, M. S.; Ruedenberg, K. Intrinsic Resolution of Molecular Electronic Wave Functions and Energies in Terms of Quasi-atoms and Their Interactions. *J. Phys. Chem. A* **2017**,

[6] Levine, D. S.; Head-Gordon, M. Energy decomposition analysis of single bonds within Kohn-Sham density functional theory. *PNAS* **2017**, *114*, 12649–12656.

[7] Schmidt, M. W.; Ivanic, J.; Ruedenberg, K. Covalent Bonds are Created by the Drive of Electron Waves to Lower their Kinetic Energy Through Expansion. *J. Chem. Phys.* **2014**, *140*, 204104.

[8] Hirshfeld, F. L.; Rzotkiewicz, M. S. Electrostatic binding in the first-row AH and A2 diatomic molecules. *Molecular Physics* **1974**, *27*, 1319–1343.

[9] Spackman, M. A.; Maslen, E. N. Chemical properties from the promolecule. *J. Phys. Chem.* **1986**, *90*, 2020–2027.

[10] Bitter, T.; Wang, S. G.; Ruedenberg, K.; Schwarz, W. H. E. Toward a physical understanding of electron-sharing two-center bonds. II. Pseudo-potential based analysis of diatomic molecules. *Theor Chem Acc* **2010**, *127*, 237–257.

[11] Fantuzzi, F.; Nascimento, M. A. C. Description of Polar Chemical Bonds from the Quantum Mechanical Interference Perspective. *J. Chem. Theory Comput.* **2014**, *10*, 2322–2332.
