# Supplementary material for: Clarifying the quantum mechanical origin of the covalent chemical bond
Source: Nat Commun. 2020 Sep 29;11:4893. doi: 10.1038/s41467-020-18670-8 (PMC7524788; doi:10.1038/s41467-020-18670-8)
Supplement: Supplementary file 3 — Descriptions of Additional Supplementary Files [file 41467_2020_18670_MOESM3_ESM.pdf]

Descriptions of Additional Supplementary Files

Supplementary Data

Description: Excel spreadsheet containing all energy decomposition analysis, and kinetic energy decomposition analysis data. The origin of the covalent H-H bond is understood as driven by kinetic energy lowering. Here the authors show this is not the case for bonds between heavier elements likely due to the presence of core electrons, and that constructive wavefunction interference instead drives bond formation.
